# First complete pterosaur from the Afro-Arabian continent: insight into pterodactyloid diversity

**DOI:** 10.1038/s41598-019-54042-z

**Published:** 2019-11-29

**Authors:** Alexander W. A. Kellner, Michael W. Caldwell, Borja Holgado, Fabio M. Dalla Vecchia, Roy Nohra, Juliana M. Sayão, Philip J. Currie

**Affiliations:** 1 Laboratory of Systematics and Taphonomy of Fossil Vertebrates, Departamento de Geologia e Paleontologia, Museu Nacional/Universidade Federal do Rio de Janeiro, Quinta da Boa Vista s/n, São Cristóvão, Rio de Janeiro 20940-040 Brazil; 2 Department of Earth and Atmospheric Sciences, University of Alberta, Edmonton, Alberta T6G2E9 Canada; 3 Institut Català de Paleontologia ‘Miquel Crusafont’ (ICP), C/de les Columnes, Universitat Autònoma de Barcelona, Cerdanyola del Vallès, Catalonia E08193 Spain; 4 Expo Hâqel, Hâqel, Main Road, Byblos, Mount Lebanon, 14014354 Lebanon; 5 Laboratório de Biodiversidade do Nordeste, Centro Acadêmico de Vitória, Universidade Federal de Pernambuco.Vitória de Santo Antão, Pernambuco, Brazil

**Keywords:** Palaeontology, Herpetology

## Abstract

Despite being known from every continent, the geological record of pterosaurs, the first group of vertebrates to develop powered flight, is very uneven, with only a few deposits accounting for the vast majority of specimens and almost half of the taxonomic diversity. Among the regions that stand out for the greatest gaps of knowledge regarding these flying reptiles, is the Afro-Arabian continent, which has yielded only a small number of very fragmentary and incomplete materials. Here we fill part of that gap and report on the most complete pterosaur recovered from this continent, more specifically from the Late Cretaceous (~95 mya) Hjoûla *Lagerstätte* of Lebanon. This deposit is known since the Middle Ages for the exquisitely preserved fishes and invertebrates, but not for tetrapods, which are exceedingly rare. *Mimodactylus libanensis* gen. et sp. nov. differs from the other Afro-Arabian pterosaur species named to date and is closely related to the Chinese species *Haopterus gracilis*, forming a new clade of derived toothed pterosaurs. Mimodactylidae clade nov. groups species that are related to Istiodactylidae, jointly designated as Istiodactyliformes (clade nov.). Istiodactyliforms were previously documented only in Early Cretaceous sites from Europe and Asia, with *Mimodactylus libanensis* the first record in Gondwana.

## Introduction

**Figure 1:**
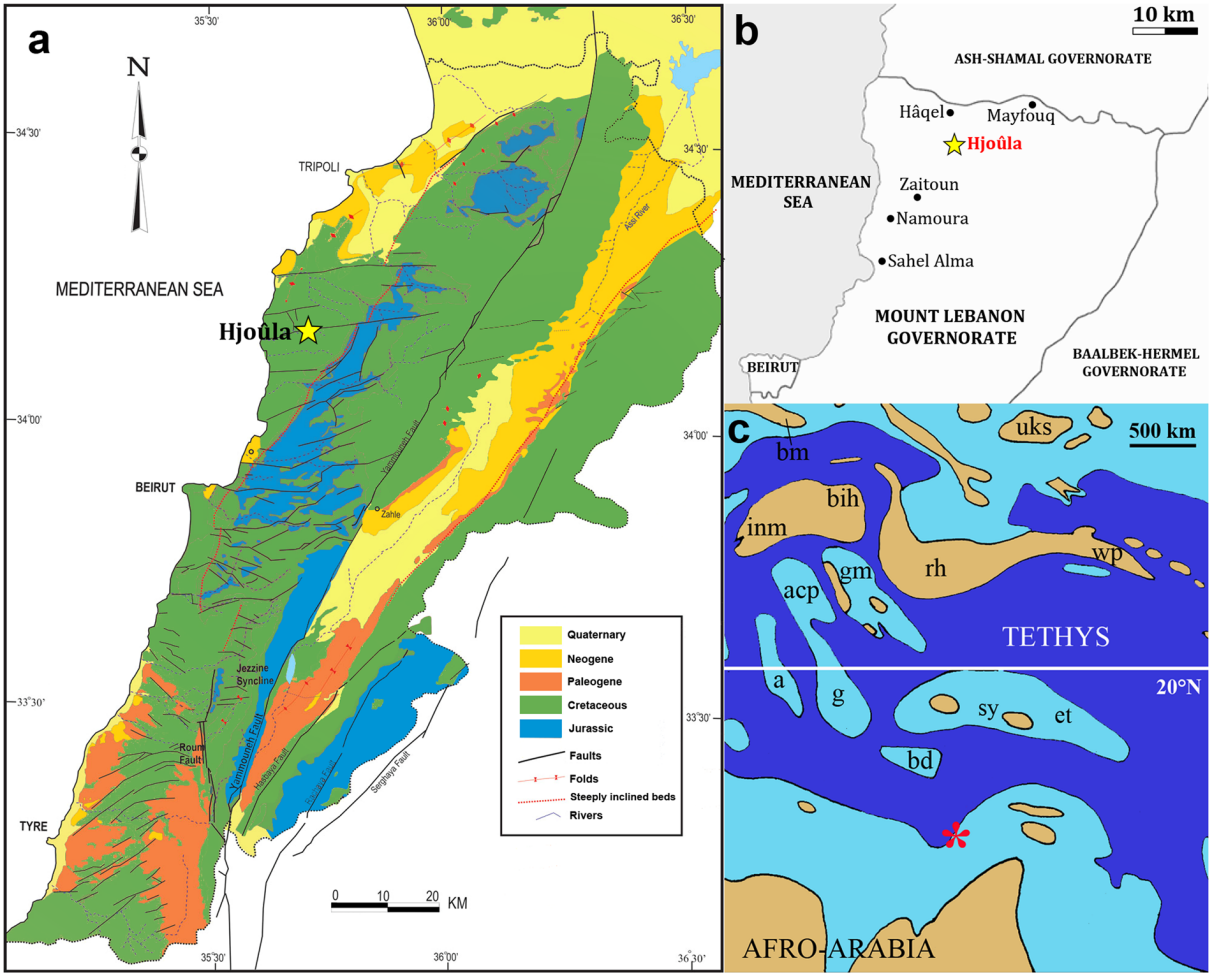
Geographical location where the new pterosaur, *Mimodactylus libanensis* gen. et sp. nov., was collected. (**a**) Geological map of Lebanon (adapted from Dubertret^14^). (**b**) Detail showing the location of the most important fossil *Lagerstätten* of Lebanon (modified from Dalla Vecchia *et al*.^65^). (**c**) Position of Lebanon in the broad carbonate platform that surrounded the northern part of the Afro-Arabian continent during the late Cenomanian (modified from Philip and Floquet^70^). Abbreviations. a = Apulian Carbonate Platform (southern Italy); acp = Adriatic Carbonate Platform (Italy, Slovenia, Croatia); bd = Bei Daglari (Turkey); bih = Bihor Massif (Romania); bm = Bohemian Massif (Central Europe); et = Eastern Taurus (Turkey); g = Gavrovo (Greece); gm = Golija Massif (Serbia); inm = Insubrian Massif (Alps); sy = Seydisehir (Turkey); uks = Ukrainian Shield (Ukraine). The yellow star indicated on each map the location of the Hjoûla *Lagerstätte*(a,b), whilst the red asterisk Lebanon(c).

Concerning Mesozoic vertebrate palaeontology, the Afro-Arabian continent is still veiled in mystery. Except for South Africa, where systematic studies, mainly in Triassic and Lower Jurassic continental deposits have provided a comparatively diverse vertebrate fauna^1^, the information about the biota that lived during most of the Mesozoic Era in this region is extremely limited. This is particularly true for pterosaurs, an extinct group of flying reptiles that includes the major powered flying vertebrates for almost 160 mya^2,3^. The main African records of this group are restricted to isolated elements from the Jurassic deposits of Tendaguru^4–6^ from Tanzania, and the Upper Cretaceous Kem Kem Beds (Cenomanian)^7–11^ and Ouled Abdoun (Maastrichtian)^12,13^ from Morocco. The most complete pterosaur specimens from the Afro-Arabian continent have been recovered from Cenomanian marine deposits of Lebanon^14^ (Fig. 1). The first specimen was a partial left forelimb of a relatively small unnamed ornithocheiroid from Hâqel *Lagerstätte*^15^ and the second a crushed skeleton formed mainly by two wings and the shoulder girdle of the azhdarchoid *Microtuban altivolans*^16^ from the coeval^14,17^ Hjoûla *Lagerstätte*.

**Figure 2:**
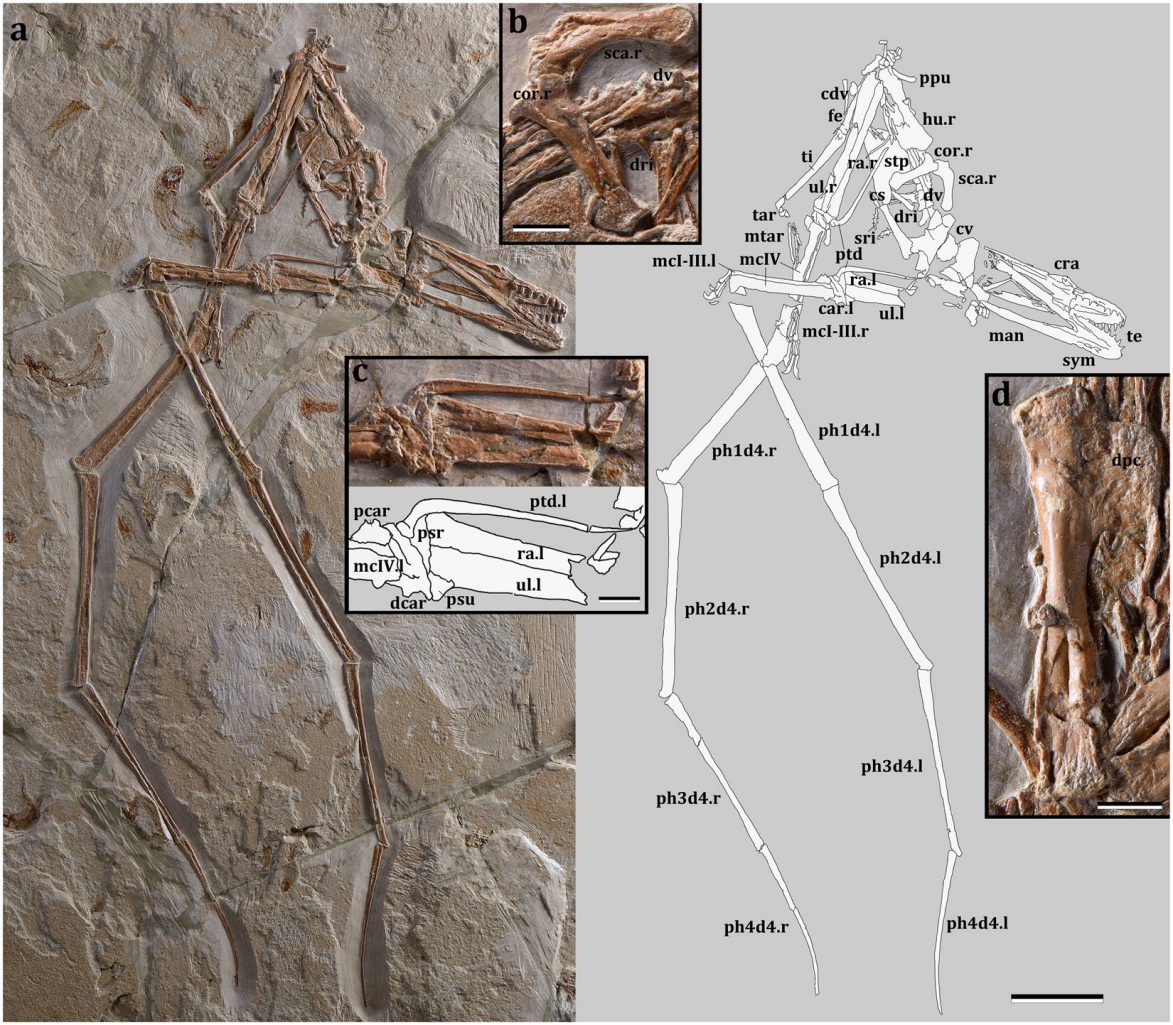
*Mimodactylus libanensis* gen. et sp. nov. (**a**) Photo and drawing of the complete specimen. (**b**) Close up of scapula and coracoid. (**c**) Detail of the wrist, showing the relation of the pteroid and the carpus. (**d**) Detail of the humerus. Scale-bars, a: 50 mm; b-d: 10 mm. Abbreviations. car: carpus; cdv: caudal vertebrae; cor: coracoid; cra: cranium; cs: cristospine; cv: cervical vertebrae; d: dentary; dcar: distal carpals; dpc: deltopectoral crest; dri: dorsal ribs; dv: dorsal vertebrae; fe: femur; hu: humerus; man: mandible; mcI-III: first to third metacarpals; mcIV: wing metacarpal; mtar: metatarsals; pcar: proximal carpals; ph1d4: first wing phalanx; ph2d4: second wing phalanx; ph3d4: third wing phalanx; ph4d4: fourth wing phalanx; ppu: prepubis; ptd: pteroid; ra: radius; sca: scapula; sri: sacral ribs; stp: sternal plate; sym: mandibular symphysis; tar: tarsus; te: teeth; ti: tibia; ul: ulna; the abbreviations ‘l’ and ‘r’ represents respectively left and right.

The specimen described here (Fig. 2) is the first complete and articulated skeleton including the skull and lower jaw from the Afro-Arabian continent providing new insights on the diversity and potential palaeoecology of ornithocheiroids.

## Results

### Systematic palaeontology

Pterosauria Kaup, 1834.

Pterodactyloidea Plieninger, 1901.

Ornithocheiroidea Seeley, 1870 *sensu* Kellner (2003)^18^.

Pteranodontoidea Marsh, 1876 *sensu* Kellner^18^.

Lanceodontia Andres *et al*.^19^.

Istiodactyliformes clade nov.

### Branch-based definition

The most inclusive clade containing *Istiodactylus latidens*, but not *Anhanguera blittersdorffi*.

### Diagnosis

Slender-built lanceodontian pterodactyloids with the following synapomorphies: mandibular rostral end pointed, teeth confined to the anterior half of the jaws, and labiolingually compressed crowns with a cingulum.

### Included taxa

Istiodactylidae, Mimodactylidae, and *Hongshanopterus lacustris*.

Mimodactylidae clade nov.

### Branch-based definition

The most inclusive clade containing *Mimodactylus libanensis* gen. et sp. nov., but not *Istiodactylus latidens*, *Ikrandraco avatar*, and *Anhanguera blittersdorffi*.

### Diagnosis

Istiodactyliforms with cone-shaped teeth, crowns with a slight labiolingual compression, and sternal articular surface of the coracoid slightly concave.

### Included species

*Haopterus gracilis* and *Mimodactylus libanensis* gen. et sp. nov.

*Mimodactylus libanensis* gen. et sp. nov.

### Etymology

*Mimodactylus*, from the acronym MIM (Mineral Museum) for the museum housing the specimen, in recognition of both the museum and the wishes of the anonymous philanthropist who facilitated the acquisition of the specimen thus keeping it in Lebanon, and the Greek ‘dactylos’ (δάκτυλος), meaning digit; *libanensis* from Lebanon, where the specimen was found.

### Holotype

Almost complete skeleton, including the skull and lower jaw, housed at the Mineral Museum (MIM) of Beirut, Lebanon, MIM F1. Cast at the University of Alberta, Edmonton and Museu Nacional/UFRJ (MN 7216-V).

### Locality and horizon

Hjoûla *Lagerstätte* of the Sannine Limestone (late Cenomanian^17^), near the town of Hjoûla located 35 km NNE of Beirut and 10 km inland from Jbail, Lebanon.

### Diagnosis

Mimodactylid with the following autapomorphies: humerus with a rectangular deltopectoral crest; humerus smaller than half the length of the second phalanx of the wing finger (hu/ph2d4 < 0.5). This species can be further distinguished from other ornithocheiroid pterodactyloids by the following combination of characters: discrete palatal ridge; 11 and 10 cone-shaped teeth on each side of the upper and lower jaws, respectively; scapula slightly longer than coracoid; humerus much longer than femur (hu/fe ~1.3); deltopectoral crest extends for around 40% of the humerus shaft length (see Supplementary Information for further details and measurements).

### Comparative description

**Figure 3:**
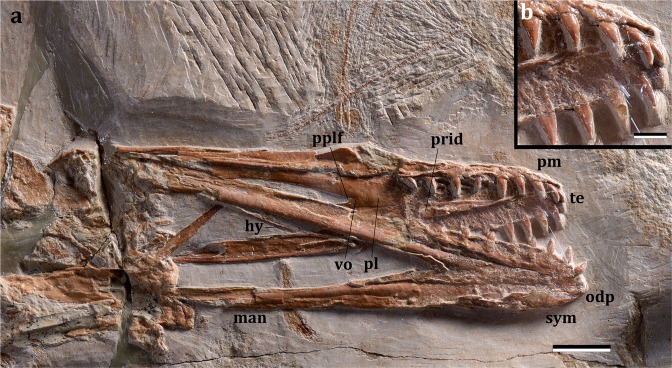
*Mimodactylus libanensis* gen. et sp. nov. (**a**) Skull and lower jaw. (**b**) Detail of the dentition. Scale bars, a: 10 mm, b: 1 mm. Abbreviations. hy: ceratobranchial I of the hyoid apparatus; man: mandible; odp: odontoid process; pl: palatine; pm: premaxilla; pplf: postpalatine fenestra; prid: palatal ridge; sym: mandibular symphysis; te: teeth; vo: vomer. Arrows point to the cingulum at the base of the teeth.

The specimen is well preserved with most parts of the skeleton articulated or only slightly displaced from their anatomical position (Fig. 2a). The skull and lower jaw are exposed in ventral view, with the occipital region and the craniomandibular articulation flattened. It is a comparatively small individual, with a wingspan of ~1.32 meters, and long wings, resulting in a high aspect ratio. Based on the unfused scapula and coracoid, pelvic elements and sacral vertebrae but fused dentaries at the symphysis, and dorsal vertebrae not fused into a notarium, it is likely that it was a very young animal at the time of death, having reached an ontogenetic stage between 2 and 3^20^. The skull has a broad rostrum in dorsopalatal view (Fig. 3), but not as rounded as in *Istiodactylus*^21–23^ and also differing from other istiodactyliforms^24,25^. The rostral tip is pointed, unlike the rounded terminus of *Istiodactylus*^21–23^. There are 11 and 10 cone-shaped teeth on each side of the upper and lower jaws, respectively (Fig. 3a), similar to *Haopterus*^26^ and *Linlongopterus*^27^. Crowns are labiolingually compressed with a cingulum (Fig. 3b; SI) as in *Haopterus* and other istiodactyliforms. This cingulum was previously reported in Istiodactylidae^28^ and related taxa, but the teeth of this latter lineage is characterised by wide crowns, which have also a marked labiolingually compression^21–25^. The palate is concave and shows a small palatal ridge. Choanae are large and divided by the vomers. The postpalatinal fenestra has an elongated egg-like shape as in the basal istiodactyliform *Hongshanopterus*^25^. Ceratobranchials I of the hyoid apparatus are fork-like, thin, and elongate elements. An odontoid process is present at the tip of the lower jaw as in *Istiodactylus latidens*^23^, but this process could also be recognised in *Haopterus*^26^ and *Lonchodraco giganteus*^29^. The dorsal vertebrae (Fig. 2b; Supplementary Fig. S1) are exposed in ventral view and are not fused into a notarium. A total of 7 caudal vertebrae were identified (Supplementary Fig. S2), all of which lack a duplex centrum and decrease rapidly in size posteriorly, suggesting that this species had a short tail. The cristospine of the sternum is comparatively short and deep, similar to those of *Nurhachius* and *Istiodactylus*; the anterior portion of the sternum is more rounded in lateral view than that of istiodactylids, being, in this respect, more similar to that of the Anhangueridae. The scapula is stout and shares with istiodactylids and anhanguerids a constricted shaft (Fig. 2b). However, it differs from both by being longer than the coracoid^18^. The coracoid sternal articulation is slightly concave as in *Haopterus*^25^ and has a developed posterior expansion that is not present in istiodactylids. The humerus (Fig. 2d) has a rectangular deltopectoral crest with an unusual straight distal margin and extends approximately 40% down the humerus shaft, more than in any other ornithocheiroid except for *Pteranodon* and related taxa^30^. *Mimodactylus* has some wing elements longer relative to the humerus compared to istiodactylids, in particular the first and second phalanges. The distal portion of the last phalanx of the wing finger is curved as in most pterosaurs. The feet are relatively small, similar to istiodactylids^30^. The pteroid (Fig. 2c) is quite large (longer than the humerus). This bone is clearly articulated with the proximal syncarpal and directed towards the body. There has been a long discussion about the position of this unique pterosaur bone with the carpal elements^31–33^, which is clearly settled in the present specimen whose forelimb bones are perfectly articulated.

## Discussion

Despite the incompleteness of the two other pterosaur specimens described from the upper Cenomanian of Lebanon, both of which lack cranial elements, those specimens can clearly be distinguished from *Mimodactylus*. The only complete bones of the ornithocheiroid MSNM V 38818 are the wing metacarpal and the radius + ulna, whose proportions differ from those of *Mimodactylus* showing that the latter has a proportionally longer forearm. Furthermore, the diameter of the radius of MSNM V 3881 is less than half that of the ulna, contrary to the condition of *Mimodactylus*. The second specimen, the holotype of *Microtuban altivolans*^16^, has a much shorter wing, a humerus with a different deltopectoral crest and a scapula that lacks the constricted shaft observed in *Mimodactylus*.

**Figure 4:**
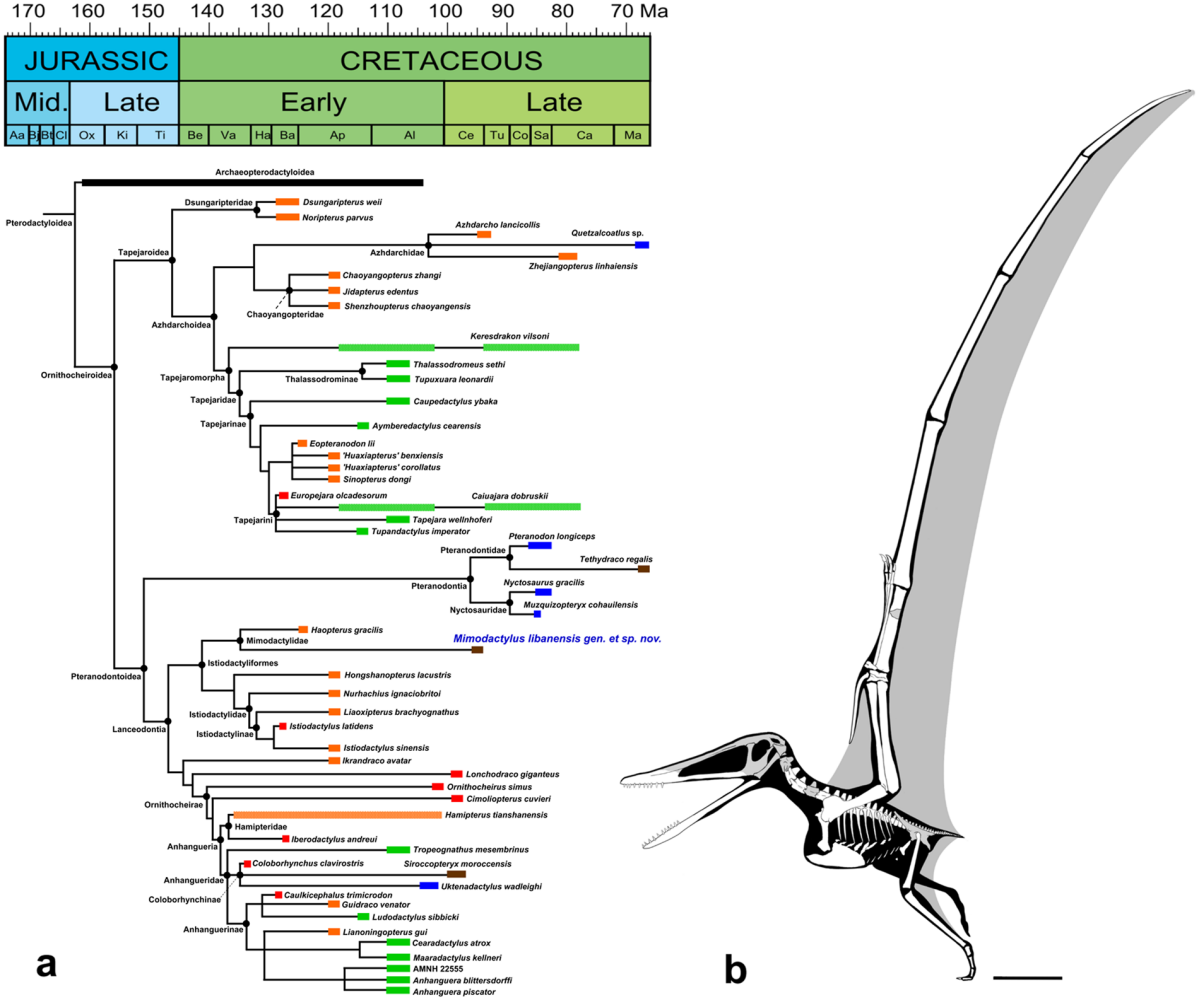
An overview of *Mimodactylus libanensis* gen. et sp. nov. (**a**) Phylogenetic relationships of *Mimodactylus libanensis* gen. et sp. nov. within Ornithocheiroidea. Colours show their continental origin: Afro-arabia (brown), Asia (orange), Europe (red), North America (blue), and South America (green). Outgroup relationships are not shown (see Holgado *et al*.^34^ and Supplementary Information for further details). Intermittent bars show uncertain temporal range. Stratigraphic chart modified from Cohen *et al*.^71^. (**b**) Reconstructed silouette of *Mimodactylus libanensis* showing the long wings regards the body. Scale bar: 50 mm.

Another interesting feature of *Mimodactylus* is the dentition (Figs. 3a and SI), which differs from that of most ornithocheroids. As in *Haopterus*^25^ and *Linlongopterus*^26^, the new species has cone-shaped dental crowns and they are confined to the anterior half part of the jaws. Such a configuration is present in other istiodactyliforms and cannot be established in *Lonchodraco giganteus* due to preservation^29^, which also present cone-shaped teeth. As in *Haopterus* but unlike *Linlongopterus* and *Lonchodraco*, teeth are characterised by a cingulum at the base of the crown (Supplementary Fig. S3), which is also present in the Istiodactylidae and closely related species. *Mimodactylus*, however, lacks the lancet-shaped teeth with marked labiolingually compressed crowns that are diagnostic of the istiodactylids^20–24^. The new species also lacks the sharp carinae reported in *Istiodactylus*^21^. The first upper tooth of *Mimodactylus* is comparatively small and has a sub-circular transverse section. It is followed by the largest teeth in the upper jaw, which have slight labiolingually compressed crowns with a cingulum, convex labial surfaces and thin, lingually inclined, needle-like tips. This general morphology is present in the remaining teeth, also from the lower jaw. This kind of dentition is more similar to that of the basal archaeopterodactyloids^18^ *Pterodactylus* and *Germanodactylus rhamphastinus*^24^ than to istiodactylids and ornithocheiroids. The sole other derived pterodactyloid with a comparable dentition is *Haopterus gracilis*, first regarded as an archaeopterodactyloid^26^, later as an ornithocheroid close to Istiodactylidae^28^, and even the sister taxa of Ornithocheiroidea^13,19^. Here we recovered *Haopterus* at the base of Laceodontia as in more recent phylogenetic analyses^34,35^, forming a clade with *Mimodactylus libanensis* (Fig. 4a; see Supplementary Information for further details).

The Mimodactylidae clade nov. is diagnosed by the following synapomorphies: cone-shaped teeth on each side of the upper jaws, crowns with a slight labiolingual compression, and sternal articular surface of the coracoid slightly concave. In addition, all mimodactylids have the teeth confined to the anterior half of the jaws and are widely spaced. Although several characters presented previously are recorded in mimodactylids and other istiodactyliforms, some of them are recovered as symplesiomorphies shared by other lanceodonts (e.g., character state 77(1): presence of an odontoid process in the lower jaw; character state 95(1): cone-shaped teeth) or are of unclear origin (e.g., character state 15(3): elongated egg-shaped postpalatine fenestra; character state 56(3): quadrate inclined about 150° posteriorly to ventral margin of the skull). In addition, two synapomorphies that support the Mimodactylidae should be considered with caution due to the missing data in most of lanceodontians^30^ (see Supplementary Information for further details) and the potential of ontogenetic variation in derived pterodactyloids^18,36^.

Despite the inherent difficulties of establishing the diet of extinct vertebrates with no suitable modern analogues such as pterosaurs, the following feeding habits have been proposed for derived pterodactyloids^37^, mainly based on their dentition (or absence of teeth) and the shape of their rostra: piscivory for Anhangueridae and their kin^30,38–40^, *Ikrandraco*^41^, Pteranodontidae^30^, Nyctosauridae^42^, Chaoyangopteridae^43^, and Thalassodrominae^44^; frugivory for Tapejarinae^45,46^; durophagy for *Dsungaripterus* and related species^30^; insectivory for *Nemicolopterus*^47^; scavenging for *Istiodactylus*^21,22^; cutting or ploughing through unconsolidated sediments for *Argentinadraco*^48^, and terrestrial stalking for the long-necked azhdarchids^49^. The dentition of *Mimodactylus* differs from all of them, suggesting that this lineage of derived pterodactyloid had a different feeding habit.

Studies on the shapes of teeth of extant insectivorous tetrapods emphasise that insectivorous species profit from having slimmer teeth that could be more easily used to breakdown arthropods due to the slight radius of curvature^50–53^. Within the pterosaur spectrum, the anurognathids that have well-spaced and isodont teeth have been regarded as insectivores^25,54^. Although wider, the tooth structure of *Mimodactylus* may suggest a similar feeding habit, allowing them to break up arthropod exoskeletons.

**Figure 5:**
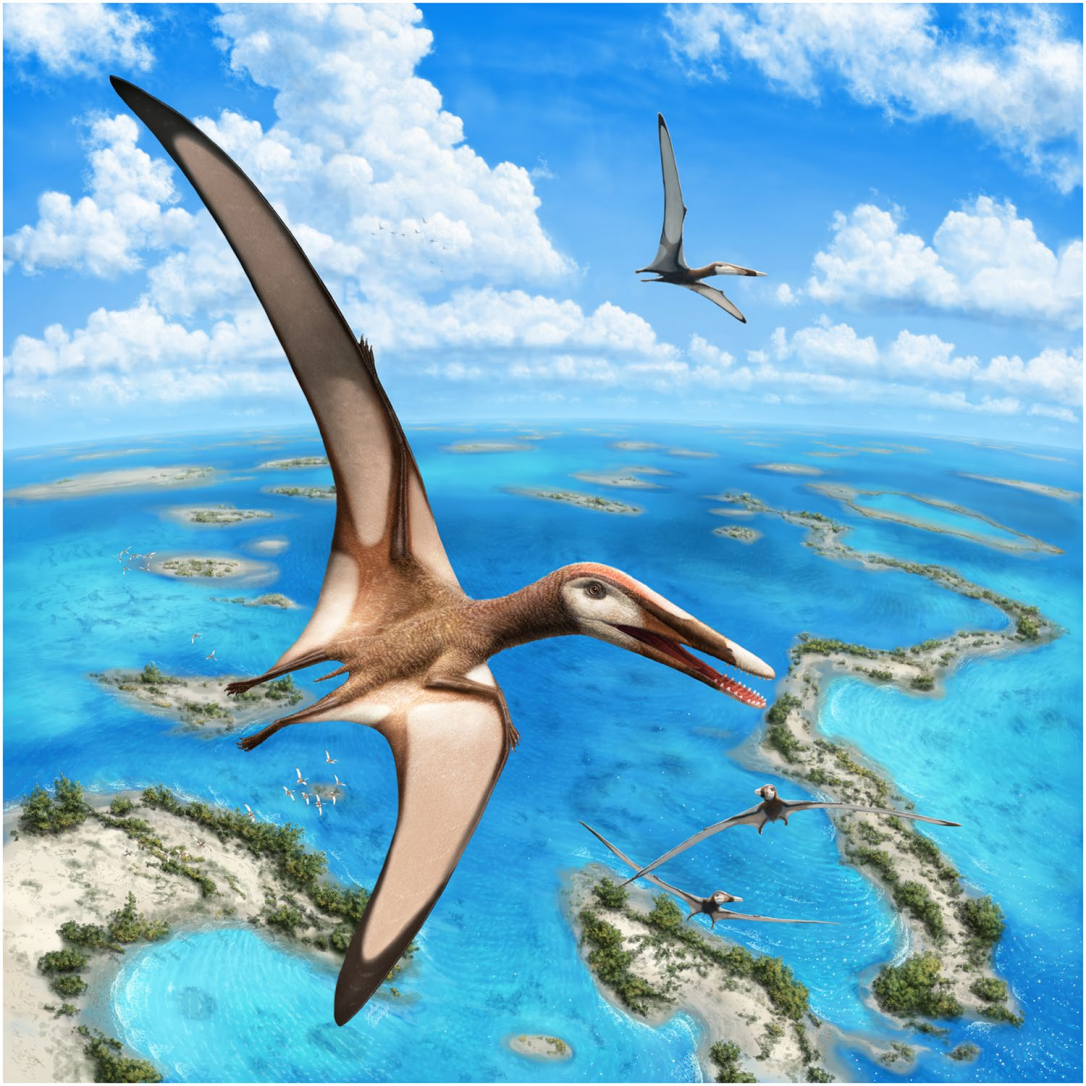
Life reconstruction of *Mimodactylus libanensis* gen. et sp. nov. Artwork of Julius T. Csotonyi.

Aerial insectivory is closely linked to the ability to maneuver during flight^55–57^. Extant vertebrate aerial insectivores exhibit short wings with low aspect ratios that allow them to be highly maneuverable in the air^56–58^, contrary to *Mimodactylus libanensis*, which had long wings with high aspect ratio (Fig. 4b). In *Mimodactylus*, as open-sea flyers, the ability to maneuver during flight appears to be limited and it was likely high stable during flight as observed in albatrosses and other birds (Fig. 5). This might also have been the case for some large pterosaurs such as anhanguerians, istiodactylids and pteranodontians, which are considered to have conducted a dynamic soaring^58^. Therefore, alternately to the insectivore hypothesis, *Mimodactylus* and their relatives might also have been capable of foraging for decapod crustaceans on surface waters, just like some albatross species feed upon caridean or penaeid shrimps^59^. In addition, a broad rostrum^60^ and spaced but relatively robust and pointed teeth^61,62^ could be good tools to seize shrimps in the water.

Observing the fossil content of the Hjoûla *Lagerstätte* as well as of other Cretaceous Lebanese *Lagerstätten*, no insects were recovered so far^63,64^. Even terrestrial plants are extremely rare at Hjoûla^65^, suggesting that this Lebanese *Lagerstätte* was far away from emergent areas such as islands, with the continent several hundred kilometres away. On the other hand, decapod crustaceans are the most common invertebrates found in Hjoûla^66,67^. No taphonomic bias was detected to explain the absence of insects, pointing to fishes and zooplankton as potentially the main local source of food for pterosaurs. *Mimodactylus libanensis* also has a broad rostrum, which is consistent with a faunivorous feeding habit - or primarily feeding on crustaceans - as present in extant ducks, boat-billed herons, and shoebills^68^. Although insectivory cannot be ruled out, all available evidence suggests that *Mimodactylus* was feeding on crustaceans.

This new pterosaur lived in archipelagos and scattered islands, which were present during the Late Cretaceous in the gigantic carbonate platform bordering the northern part of the Afro-Arabian continent with the Neotethys (Fig. 5). The discovery of *Mimodactylus libanensis* expands the spectrum of possible feeding strategies in derived pterodactyloids, a group of fascinating volant reptiles for which we still know very little.

## Methods

### Nomenclatural acts

This published work and the nomenclatural acts it contains have been registered in ZooBank, the proposed online registration system for the International Code of Zoological Nomenclature. The ZooBank Life Science Identifiers (LSIDs) can be resolved and the associated information viewed by appending the LSIDs to the prefix http://zoobank.org/. The LSID for this publication is urn:lsid:zoobank.org:pub:E77F30DB-D268-4C96-9849-1B65BCDDCAA3:, and the LSIDs for the new erected groups and taxa are: urn:lsid:zoobank.org:act:E33BF241-AD34-4878-AE3D-45F13A97F327 (Istiodactyliformes), urn:lsid:zoobank.org:act:A482B812-EA6A-4EDA-8986-3EFF1D7451B2 (Mimodactylidae), urn:lsid:zoobank.org:act:28DC7F4E-C3C3-4459-B3F1-FF58BBEF3A66 (*Mimodactylus*), and urn:lsid:zoobank.org:act:DCD7BFC3-0F35-4AA3-91B4-89A7604EAEB7 (*Mimodactylus libanensis*).

### Phylogenetic analysis

We performed a phylogenetic analysis using the software TNT 1.5^69^. This analysis is based essentially on Holgado *et al*.^34^ (for further details see Supplementary Information). Search for the most parsimonious trees (MPTs) was conducted via Traditional Search (TBR swapping algorithm), 10,000 replicates, random seed and collapsing trees after search.

## Supplementary information

Supplementary information
