# Supplementary material for: First complete pterosaur from the Afro-Arabian continent: insight into pterodactyloid diversity
Source: Sci Rep. 2019 Nov 29;9:17875. doi: 10.1038/s41598-019-54042-z (PMC6884559; doi:10.1038/s41598-019-54042-z)
Supplement: Supplementary file 1 — Supplementary information [file 41598_2019_54042_MOESM1_ESM.pdf]

SUPPLEMENTARY INFORMATION

First complete pterosaur from the Afro-Arabian continent: insight into pterodactyloid diversity

Alexander W. A. Kellner[1*], Michael W. Caldwell[2], Borja Holgado[1,3], Fabio M. Dalla Vecchia[3], Roy Nohra[4], Juliana M. Sayão[5] & Philip J. Currie[2]

[1]Laboratory of Systematics and Taphonomy of Fossil Vertebrates, Departamento de Geologia e Paleontologia, Museu Nacional/Universidade Federal do Rio de Janeiro, Quinta da Boa Vista s/n, São Cristóvão, Rio de Janeiro, 20940-040 Brazil. [2]Department of Earth and Atmospheric Sciences, University of Alberta, Edmonton, Alberta, T6G2E9 Canada. [3]Institut Català de Paleontologia 'Miquel Crusafont' (ICP), C/ de les Columnes, Universitat Autònoma de Barcelona, Cerdanyola del Vallès, Catalonia, E08193 Spain. [4]Expo Hâqel, Hâqel, Main Road, Byblos, Mount Lebanon, 14014354 Lebanon. [5]Laboratório de Biodiversidade do Nordeste, Centro Acadêmico de Vitória, Universidade Federal de Pernambuco.Vitória de Santo Antão, Pernambuco, Brazil. Correspondence and request for materials should be addressed to A.W.A.K. (email: kellner@mn.ufrj.br)

Supplementary Table S1. Measurements of some skeletal elements of *Mimodactylus libanensis* gen. et sp. nov. The length of the preserved part is reported for the incomplete elements; the abbreviations 'l' and 'r' represents respectively left and right.

Element (measured distance)	Measurement (in mm)
skull (length)	99 (preserved)
lower jaw (length)	105 (preserved)
Scapula (length)	29 (r)
Coracoid (length)	31 (r)
humerus (length)	52 (r)
humerus (dpc length)	16
ulna (length)	84 (r)
pteroid (length)	53 (l) / 52 (r)
1st wing phalanx (length)	114 (l) / 128 (r)
2nd wing phalanx (length)	117 (l) / 119 (r)
3rd wing phalanx (length)	103 (l) / 105 (r)
4th wing phalanx (length)	89 (l) / 92 (r)
femur (length)	~36 (preserved)
tibiotarsus (length)	60

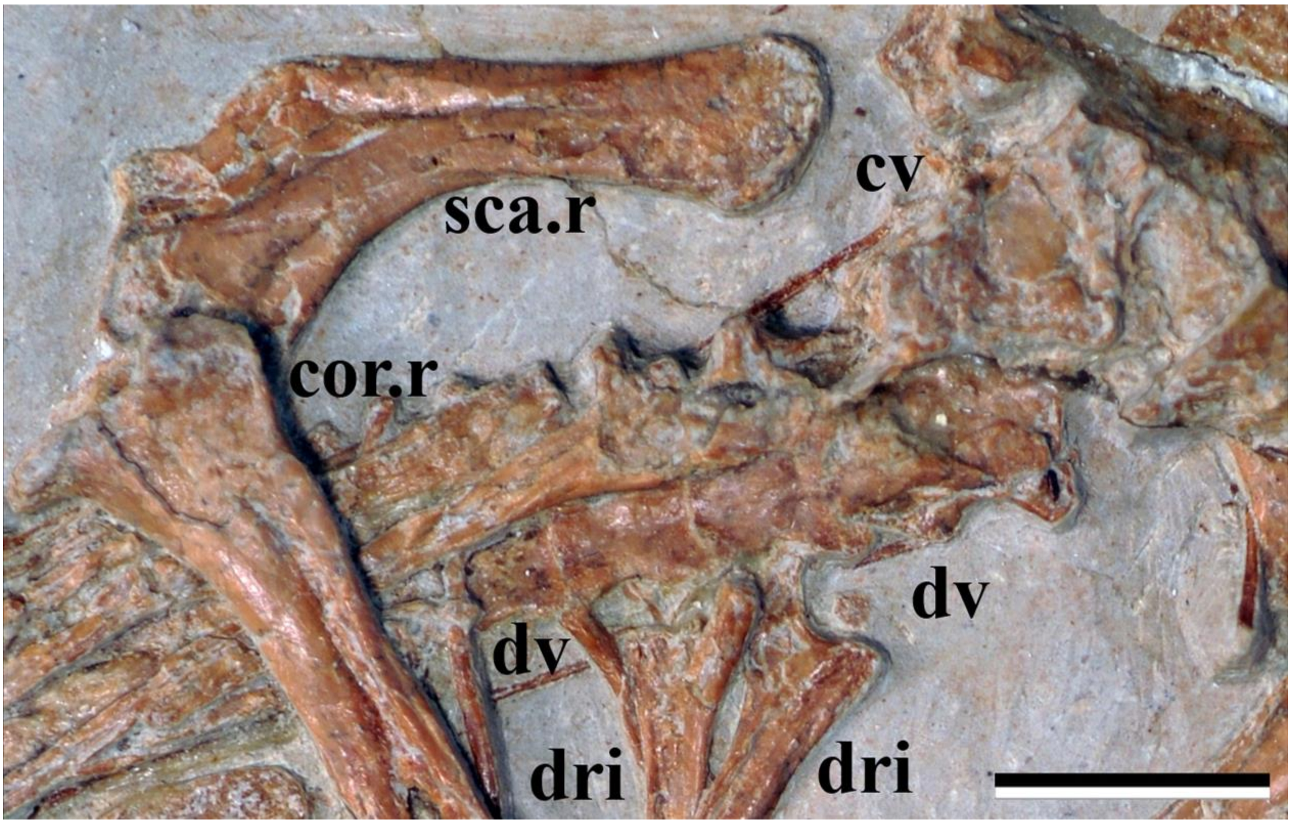

Supplementary Figure S1. Detail of the dorsal vertebrae and dorsal ribs close to the right scapula and coracoid. Scale-bar: 10 mm. Abbreviations. cor.: coracoid; cv: cervical vertebrae; dri: dorsal ribs; dv: dorsal vertebrae; l: left; r, right; sca.: scapula.

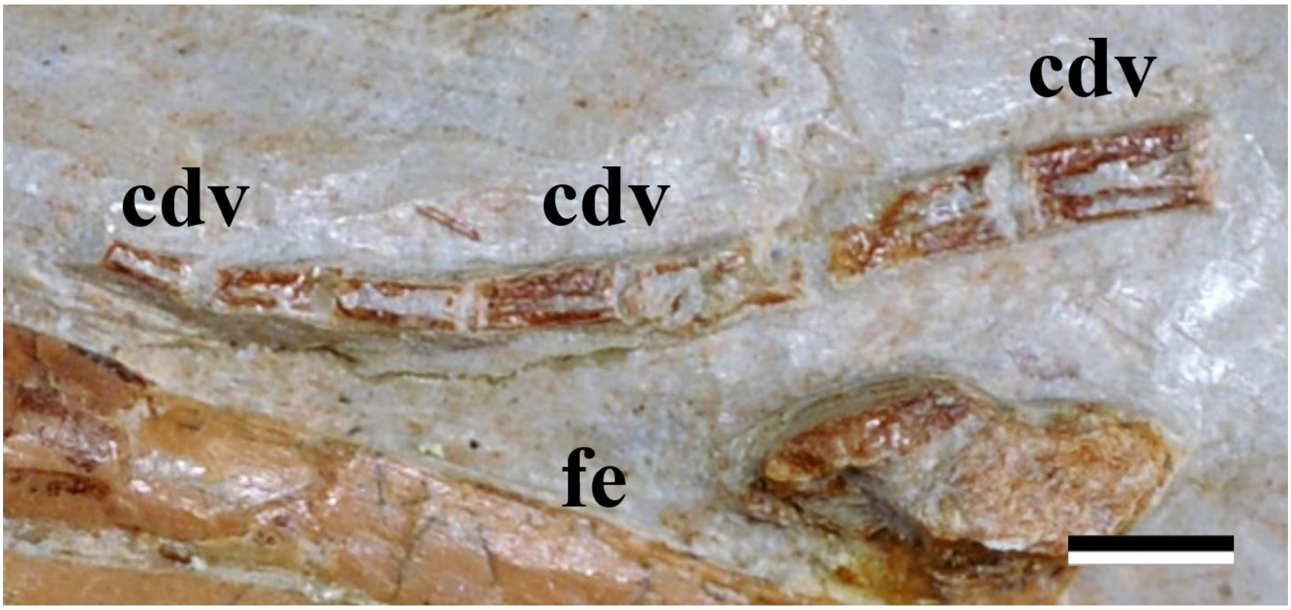

Supplementary Figure S2. Detail of the segment of caudal vertebrae. Scale-bar: 1 mm. Abbreviations. cdv: caudal vertebrae; fe: femur.

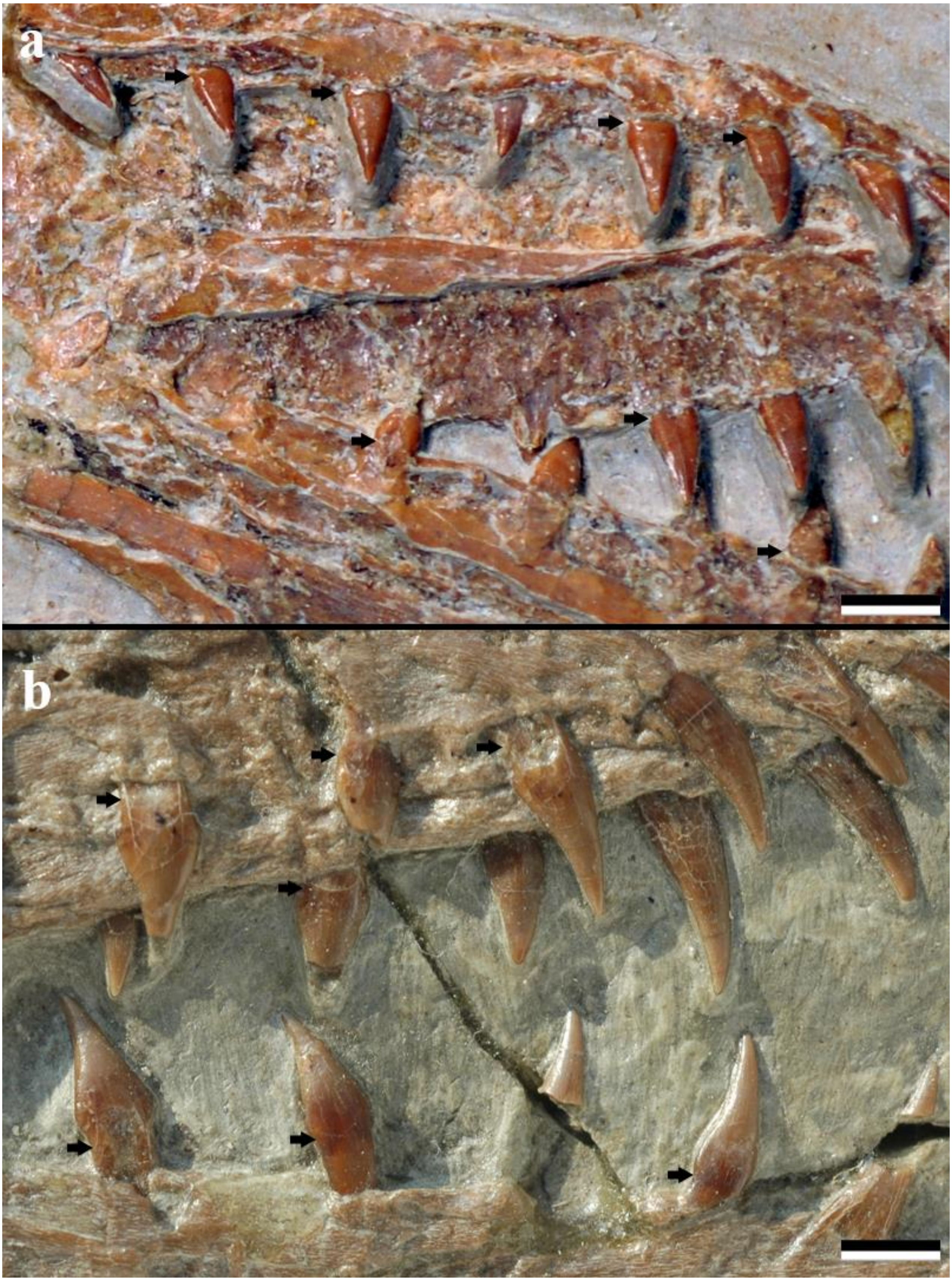

Supplementary Figure S3. Teeth comparinson of *Mimodactylus libanensis* (a) with *Haopterus gracilis* (b). Scale-bar: 1 mm. Arrows point to the cingulum at the base of the teeth.

Phylogenetic analysis

In order to establish the phylogenetic position of *Mimodactylus libanensis* gen. et sp. nov., a phylogenetic analysis was performed using TNT 1.5[69] using the TBR heuristic searches by maximum parsimony. Characters were given equal weight and treated unordered. This analysis is based essentially on Holgado et al.[34]. We added the pteranodontoid *Linlongopterus jennyae* and the istiodactylid *Liaoxipterus brachyognathus* due to the possible relationships with *Mimodactylus libanensis*, as well as the recently published tapejaromorph *Keresdrakon vilsoni*[35]. We also added five further characters: three modified from previous works[22,26-27] (characters 27, 76, and 94; for the description see the character list below) and two new characters (characters 86 and 98; see their description in the character list below). The description of character state 95(1) and character 97 have been modified respect to Holgado et al.[34]. Search for the most parsimonious trees (MPTs) was conducted via Traditional Search (TBR swapping algorithm), 10000 replicates, random seed and collapsing trees after search. The search conducted by TNT including all of the three non-pterosaur outgroups (*Ornithosuchus woodwardi*, *Herrerasaurus ischigualastensis* and *Scleromochlus taylori*) produced 12 most-parsimonious trees (MPTs) with a length of 360 steps (consistency index = 0.650; retention index = 0.871) (the strict consensus tree is shown in the Supplementary Fig. S4). Subsequently, we excluded the wildcard taxa *Linlongopterus jennyae* and run the analysis with the same parameters, obtaining six most-parsimonious trees (MPTs) with a length of 359 steps (consistency index = 0.652; retention index = 0.872) (the strict consensus tree is shown in the Supplementary Fig. S5).

An analysis via New technology was also performed using Sect. Search, ratchet (parameters: 20 substitutions made, or 99% swapping completed, six up-weighting prob., six down-weighting prob., and a total number of iterations of 10), tree fusing, Driven search (15 initial addseqs., 15 times of min. length), random seed, and without collapsing trees after search.

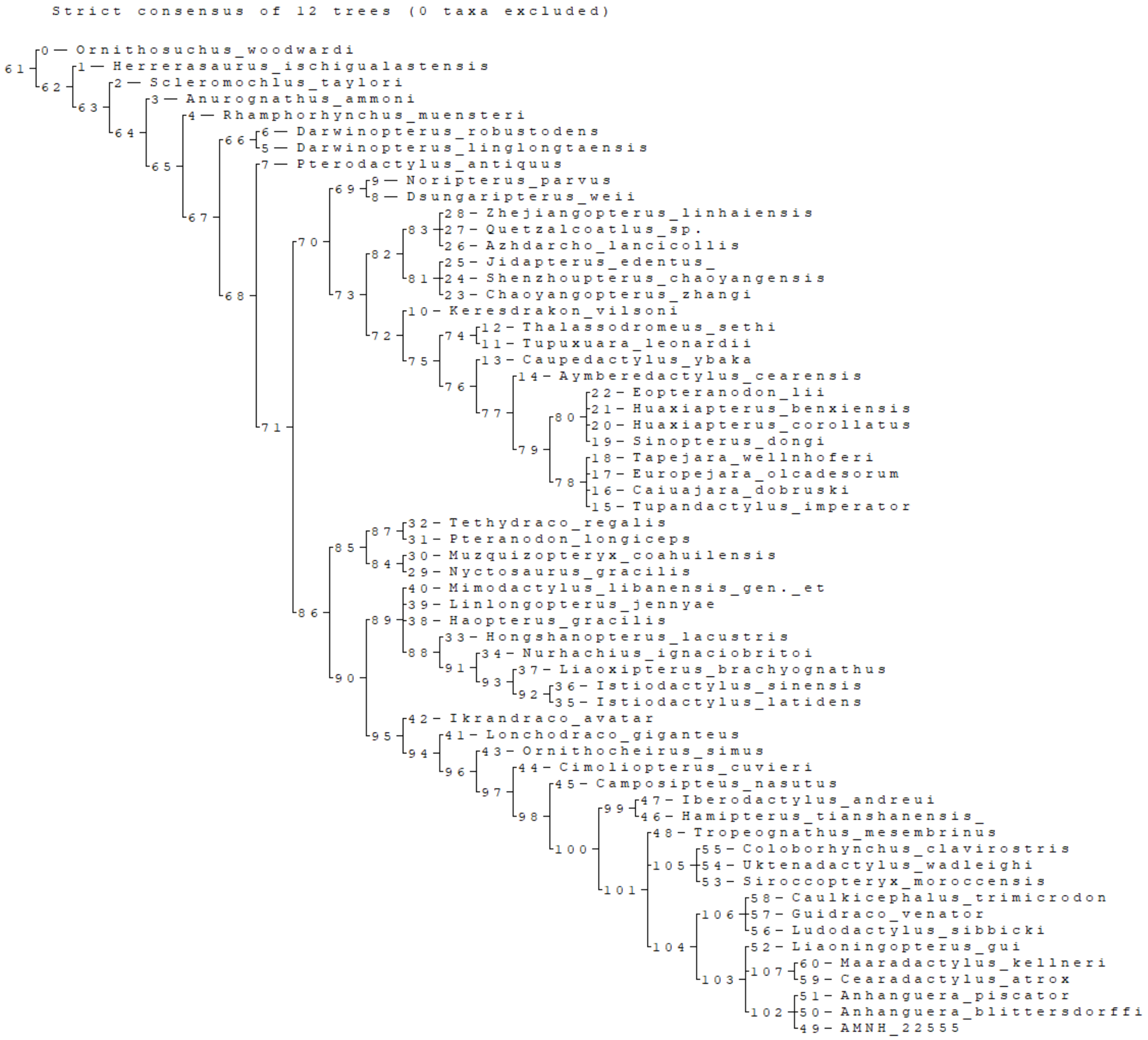

Supplementary Figure S4. Phylogenetic relationships of *Mimodactylus libanensis* gen. et sp. nov. within Pterosauria. Strict consensus of 12 most-parsimonious trees (MPTs) with a length of 360 steps (consistency index = 0.650; retention index = 0.871).

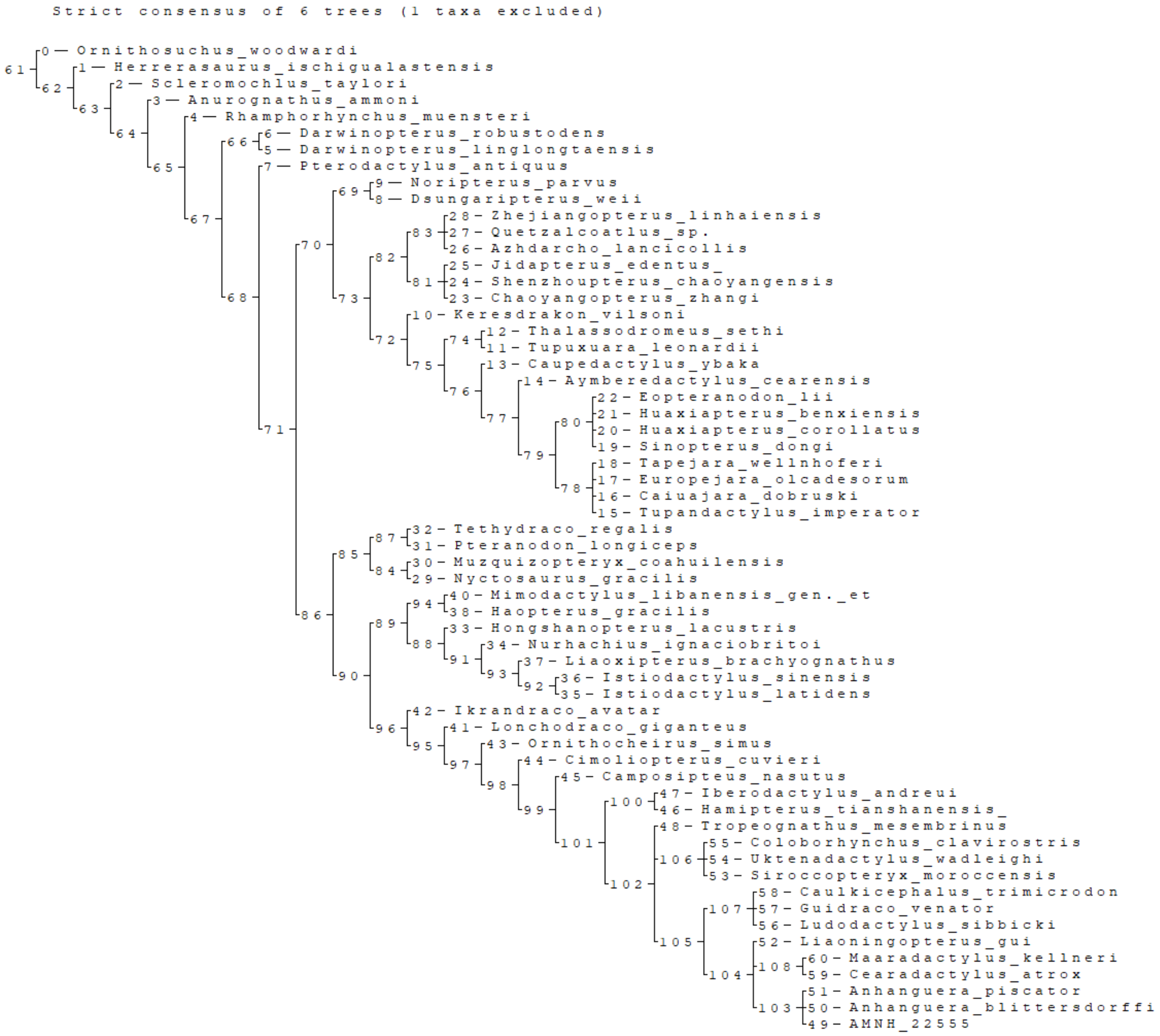

Supplementary Figure S5. Phylogenetic relationships of *Mimodactylus libanensis* gen. et sp. nov. within Pterosauria, after the exclusion of the wildcard taxon *Linlongopterus jennyae*. Strict consensus of 6 most-parsimonious trees (MPTs) with a length of 359 steps (consistency index = 0.652; retention index = 0.872).

TNT data matrix

xread

152 61

Ornithosuchus_woodwardi 0000002000001000000000000000000-00000--00--0100---

000000000000--0-0000000000?0000000--

0000000000000000000000000000000000000000000000000000000000000000

Herrerasaurus_ischigualastensis 0000100000000000000000000000-00000--00--0100---

000000000000--0-000000000000000000--

0000000000000000000000000000000000000000000000000000000000000020

Scleromochlus_taylori 0000100000000000000020000000-00000--00--0100---

00001?020??0--0-00????0000000?0000--

00000?000?000??000000???00??0000??0?000???00??0???00???00?00000000??

Anurognathus_ammoni 0000001000000000100120000000-00100--00--0110---

000110010??0--0-000000000100000000--

0000010001001000000000???0???01000?0???0??0110?1000000??10011222?0011

Rhamphorhynchus_muensteri 11001000000110011100001200000-00000--00--0100---

000000021000--0-000000001002012010--

000001000100000001000000000000010000010001111002000110 0?1001222221012

Darwinopterus_linglongtaensis 1011000000011??????0001000000-0000?20000000-

1010100010021?00--0-00?00?00????010000--

0000010001?1000000000???10??001?0?00?000?1110?100?310??1003111111022

Darwinopterus_robustodens 101100?00?011??????0001000000-0000?20000000-

1010?00010021?00--0-00?00?00????010000--

0000010001?1000000000???1???001?0?00??0??111??100?110??100311111102?

Pterodactylus_antiquus 1010000000011??0???0001000000-00000--00--00-
1001000010031?00--0-10?00000???2010000--
0000010001010000000000002110010000001000121100500?2100?1003122222020
Dsungaripterus_weii 10100001011110410200001200000-0100111000010-?---
0000100211012211011101001012012000--11012--
011000000000001111100110?000???0?2210??5?0031001??131222?2120
Noripterus_parvus 1010000101111??????0001200000-0100111000010-?---
000010021?01221101?10?00?0?2012000--?1012--
01100000000000?11??0??????????????????????0??001?????????????
Keresdrakon_vilsoni 101????????????????00?1200000-?00?0--00--?0-
???????????2????????????0000???301200101?3-------------------
?111?00?????00020011?0?01520??1????????????????
Tupuxuara_leonardii 10110020020310510210001200100-0000134000000-
?????0011002210127110110030010130120011313-------------------
11111001???00002001?21001520131001?01313???2???
Thalassodromeus_sethi 1011002002031?610210001200100-0000134000000-
1110000110022101271101100300101?0120011313-------------------
???
Caupedactylus_ybaka 1011002002031?510210021210100-0000134000000-
?????00?210221?12711011?000010130120011013-------------------
???????????00002001????015200?????????????????
Aymberedactylus_cearensis
???????????????????00??21??2012001
1?13-------------------???

Tupandactylus_imperator 1011002002031?????102121?100-0000133100000-

??????0110?22?01221101????0????20121011 2?3------------------

???

Caiuajara_dobruski 1011002002031?????1021211100-?000133100000-

???????1?00220?1221101101001??120121011213------------------

?1111000?0?00002?01?21?11520?31??120???????????

Europejara_olcadesorum

??????2???031???????0???????????????????0?????????????220?12???0???????1?1201?10

112?3------------------???

Tapejara_wellnhoferi 101100200203102????1021211100-0000133000000-

?????1011002200122110110100110120121011213------------------

?1111000?0?00002001?21011520031001 20?31????2120

Sinopterus_dongi 1011002002031?????1021211000-1000133000000-

1100010110022?11211101?0??01???20121011013------------------

?1?11000???0000200??211?152??3100?20?31222321??

Huaxiapterus_corollatus 101????????????????102121?000-1000133010000-

??????????0???????????????01????01210110?3------------------

?1?11??0???000???0??211??5???3???????31322321??

Huaxiapterus_benxiensis 1011002002031?????102121?000-1000133010000-

???????110022??1211101????01???201210110?3------------------

?1?11??0?????????????2?????????3????20?31222?2120

Eopteranodon_lii 101??0???2?????????1021211000-100013300??00-

??????????????????????????????????012001???3------------------

????????????????????21???5???2????????3?222?2???

Chaoyangopterus_zhangi 101??1?0???????????001120?000-?00????00--00-

???????????????1??????????00????012000--?3------------------

?102110????000???10?200??????3100?20?31322?21?0

Shenzhoupterus_chaoyangensis 1010012002031??????001120?000-0001155000000-

?????0??2???2?01241101????00???2012000--?3------------------

?1021??????0?0???1??200??5???3100????31222321??

Jidapterus_edentus_ 101????????????????0011200000-?001???00--00-

??????????????????????????0????3012000--13------------------

?1?2110??????0???1??200??5?0?3100????3122232120

Azhdarcho_lancicollis ???????????????????00??2?000???0?????0?--?0-

?????????????1??????????0?0??????????????3------------------

11031221???????????????015?0???????????????????

Quetzalcoatlus_sp. 10100020020????????0001200000-?00014?00--00-0---

000010??210?????????000010?3012000--13------------------

11031221?0?00002010?20?01520031001???41322?2?20

Zhejiangopterus_linhaiensis 10100020020?1??????0001200000-00000------00-0---0000100?2?00--

0-01??0?00????012000--?3------------------1??31221?0?000???10?200??5???3100?20?413???2???

Nyctosaurus_gracilis 10101000000110100100012200000-00000------00-?????00011021100--

1200?000001013012000--13------------------

11?110111000?00100013110130?03111221232222?20??

Muzquizopteryx_coahuilensis ?01010?000011???????0?????????0?00???????00-

?????0001?021?00--1200????0??????????0???3------------------

???11011????1????????11??30???111?21????????1??

Pteranodon_longiceps 10100020000111302310012200000-00000------00-

112?0000110231012311001000001013012000--03-------------------

11111011101110120001211014111311122023222232020

Tethydraco_regalis

???

??014111??????????????????

Hongshanopterus_lacustris ?????????????03011000?1100??0-?0???????--

0???????????????1??????00??0100101??????????2100000011001200000001?11101??????????????

?????????????????????????

Nurhachius_ignaciobritoi 10110020?10?1??????0001100010-00000------00-??1??01111033000-

-??????0300?0?2111000--

?22000000110012000000??111011???1?10200?2211?041??22112??1312???21??

Istiodactylus_latidens 1011000111111??????0000000010-00000------00-11100011211330?0--0-

001?0?00?011110000--

?22000000110012100000?111?011???111020002?1?10411??2112?????????????

Istiodactylus_sinensis 1011000111111??????0000000010-00000------00-?????011211330?0--0-

00?00300???111?000--

?220000001100121000001??11??1???1?1???0??21???4???2211???131222??1??

Liaoxipterus_brachyognathus

????????????????????0??0???0????1?10000

--?22?000001100120000000???

Haopterus_gracilis 1010???????????????0001100000-?0000------00-

?????0????????????????????0???12111?00--

0210010001110110000000???11??0???111?1?0?22???04???221?????3?222???20

Linlongopterus_jennyae 101000?0000?1??????0001100000-00000------00-

???????11?0311????????????0????1?11000--

?21001000101011000000???

Mimodactylus_libanensis_gen._et_sp._nov. ?????????????030??000??100000-?0??????????0-

????????????????????00??01001011111000--

?21001000111011000000???????0?00111?1?002211??4???2211?20132222221??

Lonchodraco_giganteus 1??????????????????00??100000-

?0??1??0000???????????????????????????010?????110?01?0?????1000101000000001???????????

????????????????????????????????????

Ikrandraco_avatar 10100000000110????10001100000-00000------00-11001?01000311?0--0-

00?0010010121100011 2?0000100010100?0000001111101?????????????2????41??22112201??2??

?????

Ornithocheirus_simus10??????????????????0??000?00-

?0???????????????????????????????????0?0???????????????00?0100010000001100?????????????

???????????????????????????????????

Cimoliopterus_cuvieri 1???????????????????0??000010-

?0??1?20000???????????????????????????011??????????????00?0?000???000???000????????????

???????????????????????????????????

Camposipteus_nasutus

1????????????????????0?00001100????????00???????????????????????????011??????????

????00?0?000???000???000???

Hamipterus_tianshanensis_ 10100020000?10110110001000011000000121001100-

?????001010211?0--0-001001101012010000--

10000110010000000110001111101????1?????00????10410022?12?0???????????

Iberodactylus_andreui

????????????????????0??000?110?0??1?10011??????????????????????????011??????????

????????11001000000001100??

Tropeognathus_mesembrinus 10100020000110100110 0?100001100000102000000-

?????01001021101001000100210101101000101000011?0010000001101 0?1111011???11???002?

??????10?211??????????????

AMNH_22555 10101020000110100110011000012?0000102000000-

110010101102110100100010011010 1????001?100001111010000001101011111 01?1??21112100?

???104110?2112??1??????????

Anhanguera_blittersdorffi 101010200001101001100110000120000 0102000000-

?????010110211010010001001101 01?????0101?00011110100000011010?????????????????????????

????????????????????????

Anhanguera_piscator 1010102000011?1????0011000012000001 02000000-

1100101011021101001000100?101012010001010000111101000000110101111101?10121112100

2211104110221 12??1??????2120

Liaoningopterus_gui

101?0?2000011???????0??0000120?00?1020000??????????0110211??????????0110????0?

0??1010000111101000000011010111?1???

Siroccopteryx_moroccensis

1????????????????????0??3000112?0??1??0000????????????????????????????011??????????

????0????1001000000??010???

Uktenadactylus_wadleighi

1????????????????????0??3000112?0?????0000????????????????????????????011??????????

????0????100?0?000???010??

Coloborhynchus_clavirostris

1???????????????????0??3000112?0?????0000??????????????????????????011??????????

????0????100???000???010??

Ludodactylus_sibbicki 10100000000110?????001100001??0000???000000-

11001010010211011611 00?00110??1201000101?000111101000000011110??????????????????????

?????????????????????????

Guidraco_venator 10?0000000011??????001100001??0000???000000-

?????0?0010211?1161100?00?10?0?201000?--

?00011110100000011110?1111??

Caulkicephalus_trimicrodon

10????0??0??????????01?000?12?00??1020000??????????????????11611????011??0??????

?????000??110????00????1??

Cearadactylus_atrox

10100??0010?101????0?11000?120?00?1020000?????00??1011?211?????????011010?20

10000--000011110100000001101???

Maaradactylus_kellneri

101?0?2001011???????0??0000?20?00?1020000?0?1??????0110211?1????????0110??????

0??1010000111101000000 1101???

;

cnames

{0 External_naris_(or_nasoantorbital_fenestra),_position_relative_to_the_premaxilla_

main_part_dorsal_to_the_ventral_margin_of_the_premaxilla

main_part_displaced_posterior_to_the_premaxilla;

{1 External_naris,_dorsoventrally_compressed absent present;

{2 External_naris_and_antorbital_fenestra,_configuration_ separated

confluent,_forming_a_nasoantorbital_fenestra;

{3

External_naris_and_antorbital_fenestra_(or_nasoantorbital_fenestra),_ventral_margin_length_relative_the_skull_length shorter_than_40%_of_the_skull_length_

longer_than_40%_of_the_skull_length;

{4 Antorbital_(or_nasoantorbital)_fenestra,_posterior_margin,_shape_ straight_ concave_;

{5 Nasoantorbital_(or_antorbital)_fenestra_extending_dorsal_to_the_orbit_ absent present;

{6 Orbit,_shape_ subcircular quadrangular_(broad_base) piriform_(dorsoventrally_elongated)_;

{7 Orbit,_comparatively_small absent_ present_;

{8 Ventral_margin_of_the_orbit closed open;

{9 Orbit,_position

middle_of_the_skull,_with_the_ventral_margin_of_the_orbit_below_the_middle_of_the_antorbital_(or_nasoantorbital)_fenestra_and_the_dorsal_margin_of_the_orbit_above_the_dorsal_margin_of_the_antorbital_(or_nasoantorbital)_fenestra

high_in_the_skull,_with_the_ventral_margin_of_the_orbit_the_same_level_or_above_the_middle_of_the_antorbital_(or_nasoantorbital)_fenestra

low_in_the_skull,_with_the_entire_orbit_lower_than_the_dorsal_margin_of_the_antorbital_(or_nasoantorbital)_fenestra;

{10 Suborbital_opening absent_ present_;

{11 Lower_temporal_fenestra,_shape

comparatively_broad,_with_extensive_subhorizontal_ventral_margin

piriform,_with_dorsal_portion_wider_than_ventral__

piriform,_with_ventral_portion_wider_than_dorsal_ reduced_(slit-like);

{12 Lower_temporal_fenestra,_position_relative_to_orbit posterior_to_orbit reaches_under_posterior_margin_of_orbit;

{13 Choanae,_separation separated_by_vomer confluent;

{14 Postpalatine_fenestra,_shape quadrangular/subtriangular oval egg-shaped elongated_egg-shaped_ kite-shaped,_rounded_margin elliptical reduced,_slit-like;

{15 Secondary_subtemporal_fenestra absent present;

{16 Interpterygoid_fenestra,_size smaller_than_subtemporal_fenestra larger_than_subtemporal_fenestra_ extremely_reduced;

{17 Interpterygoid_fenestra,_shape compressed_laterally broad,_longer_than_wide compressed_anteroposteriorly,_wider_than_long round;

{18 Pterygoid_fenestra absent_ present;

{19 Upper_and_lower_jaw,_marked_gap_during_occlusion absent_ present;

{20 Upper_and_lower_jaw,_shape laterally_compressed_ comparatively_broad_;

{21 Skull,_main_part_of_dorsal_margin,_curvature_excluding_cranial_crest nearly_straight concave_ convex;

{22 Length_of_the_rostrum_(pm-naof)_relative_to_the_skull_length_(pm-sq) reduced_ elongated_(about_or_less_than_half_of_skull_length)_ extremely_elongated_(more_than_half_of_skull_length);

{23 Rostrum,_anterior_end,_shape rounded pointed sharp_tip flat_surface;

{24 Rostral_end_of_premaxillae/maxillae_downturned absent_ present_;

{25 Rostrum,_distinct_concavity_on_occlusal_surface absent present;

{26 Rostrum,_anterior_portion_forming_a_high_ossified_plate absent present;

{27 Rostrum,_anterior_tip_with_a_slight_dorsal_reflection absent present;

{28 Premaxilla,_anterior_expansion absent present;

{29 Premaxilla,_anterior_expansion,_shape_in_horizantal_plane elliptical anteriorly_expanded quadrangular absent;

{30 Premaxilla,_posterior_dorsal_process,_curved_upward absent present;

{31 Premaxillae,_anterior_end_rodlike absent present;

{32 Premaxillary_process_separating_the_external_nares,_thickness_ wide narrow_;

{33 Premaxilla,_posterodorsal_margin_of_nasoantorbital_fenestra_(including_nasal),_width_ wide thin;

{34 Premaxillary_sagittal_crest absent present;

{35 Premaxillary_sagittal_crest_position confined_to_the_anterior_portion_of_the_skull_ starting_anterior_to_the_anterior_margin_of_the_nasoantorbital_fenestra,_extending_beyond_occipital_region starting_at_about_the_anterior_margin_of_the_nasoantorbital_fenestra,_reaching_the_skull_roof_above_the_orbit_but_not_extending_over_the_occipital_region starting_close_or_at_the_anterior_portion_of_the_skull_and_extended_over_the_occipital_region starting_at_the_posterior_half_of_the_nasoantorbital_fenestra starting_at_the_middle_part_of_the_nasoantorbital_fenestra_and_extended_over_the_occipital_region absent;

{36 Premaxillary_sagittal_crest,_shape striated,_low_with_a_nearly_straight_dorsal_margin striated,_high_with_a_nearly_straight_dorsal_margin round_dorsal_margin,_bladeshaped smooth,_expanded_anteriorly_and_forming_a_low_rod-like_extension_posteriorly smooth,_starting_low_anteriorly_and_very_expanded_posteriorly striated,_low,_convex_dorsal_margin;

{37 Premaxillary_crest,_elongated_dorsal_premaxillary_spike-like_projection_ absent_ present_;

{38 Premaxillary_crest,_distinct_expansion_on_the_anterior_part_ absent present;

{39 Premaxillary_crest,_concentric_striae_on_the_anterior_region absent present;

{40 Premaxillary_crest,_anterior_expansion_of_the_anterior_margin absent present;

{41 Maxilla,_posterior_ventral_expansion_ absent_ present_;

{42 Maxilla-nasal_contact absent present;

{43 Maxilla-nasal_contact,_broadness broad narrow absent;

{44 Nasal_descending_process absent present;

{45 Nasal_descending_process,_position_ placed_laterally placed_medially absent;

{46 Nasal_descending_process,_length_

long_(almost_reaching_the__ventral_margin_of_the_skull) short knob-like_(extremely_reduced)

absent;

{47 Nasal_descending_process,_orientation_ inclined_anteriorly subvertical absent;

{48 Nasal_descending_process,_lateral_foramen_ absent_ present_;

{49 Lacrimal,_extensive_fenestration absent_ present_;

{50 Lacrimal,_orbital_process_ absent_ present_;

{51 Jugal,_lacrimal_process_base,_width_ broad narrow;

{52 Jugal,_lacrimal_process,_inclination_ inclined_anteriorly subvertical inclined_posteriorly;

{53 Jugal,_presence_of_pronounced_ridge_on_the_lateral_side absent present;

{54 Jugal,_posterior_process,_orbital_process absent present;

{55 Quadrate,_inclination_relative_to_ventral_margin_of_skull_ anteriorly subvertical

inclined_about_120°_posteriorlyned_about_120°_backwards_ inclined_about_150°_posteriorly;

{56 Cranio-mandibular_articulation,_position_relative_to_orbit_

_posterior_to_posterior_margin_of_orbit under_center_of_orbit_

under_anterior_margin_of_the_orbit anterior_to_anterior_margin_of_orbit;

{57 Helical_jaw_joint absent_ present_;

{58 Frontal,_anterior_portion_rugose absent present;

{59 Frontal,_ossified_crest absent present;

{60 Frontal,_ossified_crest,_position_ confined_to_posterior_end_of_skull starting_above_orbit starting_on__posterior_half_of_nasoantorbital_fenestra absent;

{61 Frontal,_ossified_crest,_shape low,_blunt short._spike-like,_dorsally_deflected spike-like,_directed_posteriorly narrow,_broad,_directed_posteriorly low,_broad_base,_fans-shaped high,_broad_base,_crown-shaped high,_broad_base,_casqued-shaped high,_broad,_directed_posteriorly,_at_least_doubling_shight_of_skull_above_orbit absent;

{62 Parietal,_ossified_crest absent present;

{63 Parietal,_ossified_crest,_shape_ blunt_ constituting_the_base_of_the_posterior_portion_of_the_cranial_crest expanded,_with_rounded_margin absent;

{64 Posterior_region_of_the_skull_rounded_with_the_squamosal_displaced_ventrally absent_ present_;

{65 Supraoccipital does_not_extend_backwards_ extends_backwards_;

{66 Supraoccipital,_foramen_ absent_ present_;

{67 Paroccipital_processes,_expanded_distal_ends_ absent_ present_;

{68 Foraminae_piercing_the_anterior_portion_of_the_palate,_numerous absent present;

{69 Palatal_occlusal_surface smooth discrete_palatal_ridge,_tapering_anteriorly_ strong_palatal_ridge,_tapering_anteriorly strong_palatal_ridge,_confined_to_the_posterior_portion_of_the_palate;

{70 Palate,_dorsal_deflection absent present;

{71 Palate,_slight_expansion_close_to_the_anterior_margin_of_the_nasoantorbital_(or_naris_+_antorbital)_fenestra absent present;

{72 Maxilla_and_internal_naris,_contact_ absent_ present_;

{73 Palatines,_shape_ broad thin_bars;

{74 Basisphenoid_body,_length_ shorter_than_wide longer_than_wide;

{75 Mandibular_rostral_end,_extension_of_the_contact_surface_of_opposing_dentaries_ short,_limited_to_the_tip short,_extended_posteriorly_less_than_30%_of_mandible_length long,_up_to_55%_the_mandible_length long,_extended_over_55%_of_mandible_length;

{76 Mandibular_rostral_end,_odontoid_process absent present;

{77 Mandibular_rostral_end,_opposing_dentaries_ unfused fused;

{78 Mandibular_rostral_end,_shape rounded pointed sharp_tip;

{79 Dentary,_dorsal_margin,_distinct_posterior_eminence_close_to_the_separation_of_mandibular_rami absent present;

{80 Tip_of_the_dentary_projected_anteriorly absent_ present_;

{81 Dentary_ossified_sagittal_crest absent present;

{82 Dentary_ossified_sagittal_crest,_position_ confined_to_the_anterior_third_of_the_lower_jaw extinding_close_to_the_middle_portion_of_the_lower_jaw absent;

{83 Dentary_ossified_sagittal_crest,_shape_ shallow blade-like deep,_broad_in_lateral_view elongated_ridge absent;

{84 Dentary,_posteroventral_fossa absent present;

{85 Teeth,_position_and_presence present,_evenly_distributed_along_the_jaws_ teeth_absent_from_the_anterior_portion_of_the_jaws_ confined_to_the_anterior_part_of_the_jaws jaw_toothless;

{86 Teeth,_confined_to_the_anterior_part_of_the_jaws absent confined_to_the_anterior_half_of_the_jaws confined_to_the_anterior_quarter_of_the_jaws;

{87 Maxillary_teeth,_largest_positioned_posteriorly_ absent_ present_;

{88 Teeth,_shape_variation isodont heterodont;

{89 Teeth,_anterior,_height_versus_width_proportion more_than_twice_their_width less_than_twice_their_width;

{90 Teeth,_anterior,_marked_variation_in_size absent present;

{91

Teeth,_upper_jaw,_variation_in_the_size_of_the_anterior_teeth_with_the_5th_and_6th_smaller_than_the_4th absent_ present_;

{92 Teeth,_base_broad_and_oval absent_ present_;

{93 Teeth,_serrated present absent;

{94 Teeth,_cingulum absent present;

{95 Teeth,_peg-like_(cone-shaped) absent_ present;

{96 Teeth,_small_needle-shaped absent present;

{97 Teeth,_labiolingually_compressed_crowns absent present;

{98 Teeth,_labiolingually_compressed_crowns,_compression not_compressed slightly_compressed strongly_compressed;

{99 Teeth,_sharp_carinae absent present;

{100 Teeth,_elongated absent present;

{101 Teeth,_striated absent present;

{102 Teeth,_curvature_of_the_toothline absent present;

{103 Teeth,_first_pair_above_second_pair absent present;

{104 Alveoli,_lateral_platform absent present;

{105 Atlas_and_axis unfused_ fused_;

{106 Cervical_vertebrae,_postexapophyses_ absent_ present_;

{107 Mid-cervical_vertebrae,_centrum,_lateral_foramen_ absent_ present_;

{108 Mid-cervical_vertebrae,_length short,_sub-equal_in_length_

longer_than_wide,_with_length_less_than_3_times_width

elongated,_with_length_more_than_3_times_width extremely_elongate;

{109 Mid-cervical_vertebrae,_ribs present_ absent_;

{110 Mid-cervical_vertebrae,_neural_spines,_height_ tall low extremely_reduced_or_absent;

{111 Mid-cervical_vertebrae,_neural_spines,_shape_ blade-shaped spike-shaped_ ridge;

{112 Notarium absent_ present_;

{113 Caudal_vertebrae,_quantity_ more_than_15_ 15_or_less_;

{114 Caudal_vertebrae,_zygapophyses_forming_rod-like_ossified_processes_ absent present;

{115 Proximal_caudal_vertebrae_centrum,_centrum_shape_ single douplex;

{116 Scapula,_length_relative_to_coracoid_length subequal_or_longer_than_coracoid_

scapula_shorter_than_coracoid_(1_>_sca/cor_>_0.80)_

substantially_shorter_than_coracoid_(sca/cor_<_0.80);

{117 Scapula,_proximal_end_ elongated_ sub-oval_;

{118 Scapula,_shape_ elongated_ stout,_with_constructed_shaft_;

{119 Coracoid,_proximal_end,_shape flattened oval;

{120 Coracoid,_sternal_articulation_ no_developed_articulation

articulation_surface_straight_or_slightly_concave articulation_surface_strongly_concave;

{121 Coracoid,_sternal_articulation,_posterior_expansion_ absent present;

{122 Coracoid,_ventral_margin,_deep_flange absent present;

{123 Coracoid,_broad_tubercle_on_ventroposterior_margin absent present;

{124 Cristospine,_shape absent_ shallow_and_elongated_ deep_and_short;

{125 Humerus,_proportional_length_relative_to_the_metacarpal_IV_(hu/mcIV)_

hu/mcIV_>_2.50_ 1.50_<_hu/mcIV_<_2.50_ 0.40_<_hu/mcIV_<_1.50 hu/mcIV_<_0.40;

{126 Humerus,_proportional_length_relative_to_the_femur_(hu/fe)_ hu/fe_<0.80_

1.4_>_hu/fe>___0.80_ hu/fe_>_1.40;

{127 Humerus_plus_ulna,_proportional_lengths_relative_to_the_femur_plus_tibia_(hu+ul/fe+ti)_

humerus_plus_ulna_about_0.80%_or_less_of_femur_plus_tibia_length_(hu+ul/fe+ti_<_0.80)_

humerus_plus_ulna_larger_than_0.80%_of_femur_plus_tibia_length_(hu+ul/fe+ti_>_0.80)_;

{128 Humerus,_proximal_end,_small_foramen_on_dorsal_surface_distal_to_proximal_articulation

absent present;

{129 Humerus,_proximal_end,_foramen_on_ventral_surface_close_to_proximal_margin_ absent_

present_;

{130 Humerus,_deltopectoral_crest,_shape_ reduced,_positioned_close_to_the_humerus_shaft

enlarged,_proximally_placed,_with_almost_straight_proximal_margin_

enlarged,_hatchet_shaped,_proximally_placed

enlarged,_hatched_shaped,_positioned_further_down_the_humerus_shaft enlarged,_warped

long,_proximally_placed,_curving_ventrally;

{131 Humerus,_medial_(=_ulnar)_crest reduced_ directed_posteriorly_

massive,_with_a_developed_proximal_ridge;

{132 Humerus,_distal_articulation,_shape_ oval_or_D-shaped_ subtriangular_;

{133 Humerus,_between_distal_condyles,_pneumatic_foramen absent present;

{134 Ulna,_proportional_length_relative_to_metacarpal_IV_(ul/mcIV)_

ulna_3.6_times_longer_than_metacarpal_IV_(ul/mcIV_>_3.6)_

length_of_ulna_between_3.6_and_two_times_the_length_of_metacarpal_IV_(3.6_>ul/mcIV>2)_

ulna_between_double_and_the_same_length_of_metacarpal_IV_(2>ul/mcIV>1)

ulna_the_same_size_or_smaller_than_metacarpal_IV_(ul/mcIV_<_1);

{135 Ulna_and_radius,_diameter_at_midshaft_ subequal_

diameter_of_radius_about_half_that_of_ulna diameter_of_radius_less_than_half_that_of_ulna;

{136 Proximal_syncarpal,_large_posterodistal_process absent present;

{137 Proximal_syncarpal,_shape_(proximal_view) quadrangular_or_irregular pentagonal;

{138 Distal_syncarpals,_shape_(distal_view)_ irregular form_rectangular_unit_ form_triangular_unit;

{139 Pteroid absent_ shorter_than_half_the_length_of_the_ulna_ longer_that_half_the_length_of_the_ulna;

{140 Pteroid,_proximal_articulation,_expanded_in_right_angle_with_shaft absent present;

{141 Metacarpals_I_-_III,_relation_with_carpus_ articulating_with_carpus_ metacarpal_I_articulates_with_carpus,_metacarpals_II_and_III_reduced_ not_articulating_with_carpus;

{142 Manual_digit_IV_first_phalanx,_proportional_length_relative_to_metacarpal_IV_(ph1d4/mcIV)_ both_small_and_reduced_ ph1d4/mcIV>4.0 4.0>ph1d4/mcIV>2.0 '2.0>_ph1d4/mcIV>1.0' 'ph1d4/mcIV<_1.0';

{143 Manual_digit_IV_first_phalanx,_proportional_length_relative_to_tibiotarsus_(ph1d4/ti)_ ph1d4_reduced_ ph1d4_elongated_and_less_than_twice_the_length_of_ti_(ph1d4/ti_smaller_than_2.00) ph1d4_elongated_about_or_longer_than_twice_the_length_of_ti_(ph1d4/ti_subequal/larger_than_2.00);

{144 Manual_digit_IV_second_phalanx,_proportional_length_relative_to_first_phalanx_(ph2d4/ph1d4)_ both_short_or_absent_ elongated_with_second_phalanx_about_the_same_size_or_longer_than_first_(ph2d4/ph1d4_larger_than_1.00)_ elongated_with_second_phalanx_up_to_30%_shorter_than_first_(ph2d4/ph1d4_between_0.70_-

_1.00)

elongated_with_second_phalanx_more_than_30%_shorter_than_first_(ph2d4/ph1d4_smaller_than_0.70);

{145

Manual_digit_IV_third_phalanx,_proportional_length_relative_to_first_phalanx_(ph3d4/ph1d4)_ both_short_or_absent_ ph3d4_about_the_same_length_or_larger_than_ph1d4_

ph3d4_shorter_than_ph1d4;

{146

Manual_digit_IV_third_phalanx,_proportional_length_relative_to_the_second_phalanx_(ph3d4/ph2d4)_ both_short_or_absent_ ph3d4_about_the_same_size_or_longer_than_ph2d4_

ph3d4_shorter_than_ph2d4;

{147

Proportional_length_of_the_forth_phalanx_of_manual_digit_IV_relative_to_the_first_phalanx_of_manual_digit_IV_(ph4d4/ph1d4) both_short_or_absent

both_elongated,_with_the_forth_phalanx_longer_than_the_first_(ph4/d4>1.00)

both_elongated,_with_the_forth_phalanx_the_same_length_or_shorter,_but_longer_than_35%_the_lenght_of_the_first

both_elongated,_with_the_forth_phalanx_less_than_35%_the_lenght_of_the_first;

{148 Femur,_length_relative_to_metacarpal_IV_length_(fe/mcIV)_

femur_at_least_twice_the_metacarpal_IV_length_(fe_?_mcIV_>_2.00)

femur_longer_but_less_than_twice_the_length_of_metacarpal_IV_(1.00_<_fe/mcIV_<_2.00)_

femur_about_the_same_length_or_shorter_than_metacarpal_IV_(fe/mcIV_<_1.00);

{149 Metatarsal_III,_proportional_length_relative_to_tibia_length_

more_than_30%_of_tibia_length_ less_than_30%_of_tibia_length_;

{150 Pedal_digit_V,_number_of_phalanges_ with_four_phalanges_ with_2_phalanges_

with_1_or_no_phalanx_(extremely_reduced);

{151 Pes,_second_phalanx_of_digit_V,_shape_ reduced_or_absent_ elongated,_straight_

elongated,_curved elongated,_very_curved_(boomerang_shape);

;

proc /;
